# The differential diagnosis of IgG4-related disease based on machine learning

**DOI:** 10.1186/s13075-022-02752-7

**Published:** 2022-03-19

**Authors:** Motohisa Yamamoto, Masanori Nojima, Ryuta Kamekura, Akiko Kuribara-Souta, Masaaki Uehara, Hiroki Yamazaki, Noritada Yoshikawa, Satsuki Aochi, Ichiro Mizushima, Takayuki Watanabe, Aya Nishiwaki, Toshihiko Komai, Hirofumi Shoda, Koji Kitagori, Hajime Yoshifuji, Hideaki Hamano, Mitsuhiro Kawano, Ken-ichi Takano, Keishi Fujio, Hirotoshi Tanaka

**Affiliations:** 1 Department of Rheumatology and Allergy, IMSUT Hospital, The Institute of Medical Science, The University of Tokyo, 4-6-1, Shirokanedai, Minato-ku, Tokyo, 1088639 Japan; 2 Center for Translational Research, IMSUT Hospital, The Institute of Medical Science, The University of Tokyo, Tokyo, Japan; 3 Department of Otolaryngology, Sapporo Medical University School of Medicine, Sapporo, Japan; 4 Division of Rheumatology, Center for Vaccine and Therapy, IMSUT Hospital, The Institute of Medical Science, The University of Tokyo, Tokyo, Japan; 5 Department of Internal Medicine, Japan Self Defense Sapporo Hospital, Sapporo, Japan; 6 Department of Rheumatology, Kanazawa University Hospital, Kanazawa, Japan; 7 Second Department of Internal Medicine, Shinshu University School of Medicine, Matsumoto, Japan; 8 Department of Allergy and Rheumatology, Graduate School of Medicine, The University of Tokyo, Tokyo, Japan; 9 Department of Rheumatology and Clinical Immunology, Graduate School of Medicine, Kyoto University, Kyoto, Japan; 10 Nagano Prefectural Kiso Hospital, Kiso, Japan

**Keywords:** Artificial intelligence, Differential diagnosis, IgG4-related disease, Machine learning

## Abstract

**Introduction:**

To eliminate the disparity and maldistribution of physicians and medical specialty services, the development of diagnostic support for rare diseases using artificial intelligence is being promoted. Immunoglobulin G4 (IgG4)-related disease (IgG4-RD) is a rare disorder often requiring special knowledge and experience to diagnose. In this study, we investigated the possibility of differential diagnosis of IgG4-RD based on basic patient characteristics and blood test findings using machine learning.

**Methods:**

Six hundred and two patients with IgG4-RD and 204 patients with non-IgG4-RD that needed to be differentiated who visited the participating institutions were included in the study. Ten percent of the subjects were randomly excluded as a validation sample. Among the remaining cases, 80% were used as training samples, and the remaining 20% were used as test samples. Finally, validation was performed on the validation sample. The analysis was performed using a decision tree and a random forest model. Furthermore, a comparison was made between conditions with and without the serum IgG4 concentration. Accuracy was evaluated using the area under the receiver-operating characteristic (AUROC) curve.

**Results:**

In diagnosing IgG4-RD, the AUROC curve values of the decision tree and the random forest method were 0.906 and 0.974, respectively, when serum IgG4 levels were included in the analysis. Excluding serum IgG4 levels, the AUROC curve value of the analysis by the random forest method was 0.925.

**Conclusion:**

Based on machine learning in a multicenter collaboration, with or without serum IgG4 data, basic patient characteristics and blood test findings alone were sufficient to differentiate IgG4-RD from non-IgG4-RD.

## Introduction

Rheumatic diseases are currently diagnosed using diagnostic criteria based on a combination of physical, hematological, imaging, and pathological findings. As such, several visits to medical institutions and invasive examinations are needed before a diagnosis can be made. This is problematic for some patients, especially during the ongoing severe acute respiratory syndrome coronavirus 2 (SARS-CoV-2) pandemic, which has prevented patients from visiting medical institutions and has exacerbated the disparity in medical care between different regions.

In particular, this problem is a major obstacle for the diagnosis of immunoglobulin (Ig) G4-related disease (IgG4-RD). IgG4-RD is a systemic fibroinflammatory disease characterized by elevated serum levels of IgG4, marked infiltration of IgG4-bearing plasma cells, and fibrosis in the involved organs [1]. It is a rare disease that was newly conceptualized in this century, and it has many differential diagnoses. General physicians do not always recognize IgG4-RD in patients. In addition, delays in diagnosis and treatment can lead to severe organ dysfunction.

The use of artificial intelligence (AI) may help solve this issue. Machine learning, which is a data analysis technique for realizing AI, is a method in which computers automatically analyze data to discover and learn the rules and patterns behind the data. In recent years, there has been an emphasis on making predictions and decisions based on the results of such learning, and the use of AI in the medical field has increased. It has been successful in building models for retrospectively identifying abnormalities in diverse types of images [2]. In particular, systems for detecting colorectal cancer, skin tumors, cerebral aneurysms, and influenza infection, among others, by AI-based imaging diagnosis have been consecutively developed. In rheumatology, many results related to treatment support have been reported. Studies have successfully predicted the response to treatment and the prognosis of rheumatoid arthritis (RA) patients using data on clinical markers and genetic analyses. In 2019, Kim et al. used transcriptome profiling of RA synovium to predict treatment responses from inflammatory signals [3]. Furthermore, Guan et al. used clinical data and single-nucleotide polymorphism sequence data to predict the patient response to antitumor necrosis factor therapy [4]. Subsequently, many prognostic predictors of the patient response to treatment and rehospitalization have been reported for RA, systemic lupus erythematosus (SLE), juvenile idiopathic arthritis, spondyloarthropathy, and osteoarthritis. However, there have not yet been any AI studies for IgG4-RD.

The diagnosis of IgG4-RD is made based on physical, imaging, serological, and histopathological findings [5]. For this reason, the diagnosis requires the judgment of many specialists. However, ever since the Fukushima nuclear accident in 2011 in Japan, patients have become very nervous about radiation exposure, including that from diagnostic tests, and the current SARS-CoV-2 pandemic had prevented the diagnosis of IgG4-RD in many patients. Therefore, to facilitate the diagnosis of IgG4-RD, in this study, we attempted to determine whether AI could properly diagnose IgG4-RD using only physical examination and blood test data obtained at the first visit, which are available to general practitioners, without using images and pathological findings that require a diagnosis by specialists.

In addition, the therapeutic strategy for rheumatic diseases is decided after carefully considering the distribution and degree of disability. These are the areas in which AI excels the most. Currently, the diagnosis of IgG4-related disease (IgG4-RD) is based on blood test results; findings from imaging examinations such as computed tomography (CT), MRI, and fluorodeoxyglucose positron emission tomography (FDG-PET), and histopathological findings. As a result, the invasiveness to the patients is high, and the high cost of medical care has become a problem. Thus, this study investigated whether AI can be trained to differentiate IgG4-RD from other rheumatic diseases by learning the typical cases of both IgG4-RD and non-IgG4-RD, and whether proper diagnosis is possible. The results of this study are expected to be useful for assisting nonspecialist physicians in the community to make appropriate diagnosis and treatment decisions for patients with IgG4-RD.

## Methods

### Patients

The subjects were 602 patients with IgG4-RD who visited the Institute of Medical Science, The University of Tokyo (IMSUT) Hospital, The University of Tokyo Hospital, Kanazawa University Hospital, Shinshu University Hospital, Kyoto University Hospital, or Sapporo Medical University Hospital between April 1997 and June 2019. In addition, 204 patients with other rheumatic disorders that needed to be differentiated from IgG4-RD who visited the aforementioned institutions between January 2019 and June 2019 were also included in the study. All subjects provided informed consent to participate in the study based on the information provided in the study. IgG4-RD was diagnosed based on the comprehensive diagnostic criteria for IgG4-RD (Ministry of Health, Labour and Welfare Research Group, Japan, 2011) [6]. Among those who visited the aforementioned institutions before 2010, only those with a definitive diagnosis according to the comprehensive diagnostic criteria were included in this study. The differential diseases were as follows: Sjögren’s syndrome (SS; *N*=106), SLE (*N*=25), polymyositis/dermatomyositis (*N*=19), microscopic polyangiitis (*N*=6), eosinophilic granulomatosis with polyangiitis (EGPA; *N*=11), granulomatosis with polyangiitis (*N*=10), multicentric Castleman’s disease (MCD; *N*=19), and sarcoidosis (*N*=8). In actual clinical practice, the differential diagnosis of IgG4-RD is very important. Hyper-IgG4emia is not specific to IgG4-RD as it is also detected in eosinophilic diseases, such as EGPA, eosinophilic pneumonia, some bronchial asthma, and MCD [7]. It is also necessary to differentiate IgG4-RD from other disorders, including SS in sicca symptoms, SS and sarcoidosis in lacrimal and salivary gland swelling, SLE in hypocomplementemia, and myositis and vasculitis in interstitial pneumonia. Depending on the diagnosis, the treatment and prognosis may vary greatly. However, because IgG4-RD is a rare disease, the differential diagnosis can be difficult. In this study, the diagnostic or classification criteria developed by the relevant societies and research groups were used [8–15].

### Machine learning and statistical analyses

First, a dataset including two basic patient characteristics and 29 laboratory findings from the first visit was created for each case (Table 1). Ten percent of the patients and controls were randomly selected to be validation samples. Of the remaining cases, 80% and 20% were included as training and test samples, respectively [16]. This process was performed randomly by a computer for each of the IgG4-RD and non-IgG4-RD groups. Finally, validation was performed with the validation sample that was separated at the beginning. A prediction model was developed using the training sample with the outcome set to extract IgG4-RD cases from among the cases of diseases that needed to be differentiated from IgG4-RD. We also performed analyses for the situations with known and unknown serum IgG4 concentration, which are considered to be an important variable in the diagnosis of IgG4-RD. In the latter situation, we purposely excluded the IgG4 concentration from the analysis. Cases with missing data were excluded from the study. The value of missing data was 0.7%. R version 3.6.1 software (https://cran.ism.ac.jp) was used for the analyses. The following classification and regression tree (CART) and random forest models were applied using the R package “rpart” (v. 4.1-15) (https://cran.ism.ac.jp/bin/macosx/contrib/4.0/rpart_4.1-15.tgz) and “randomForest”(v. 4.6-14) (https://cran.ism.ac.jp/bin/macosx/contrib/4.0/randomForest_4.6-14.tgz), respectively.

**Table 1:**
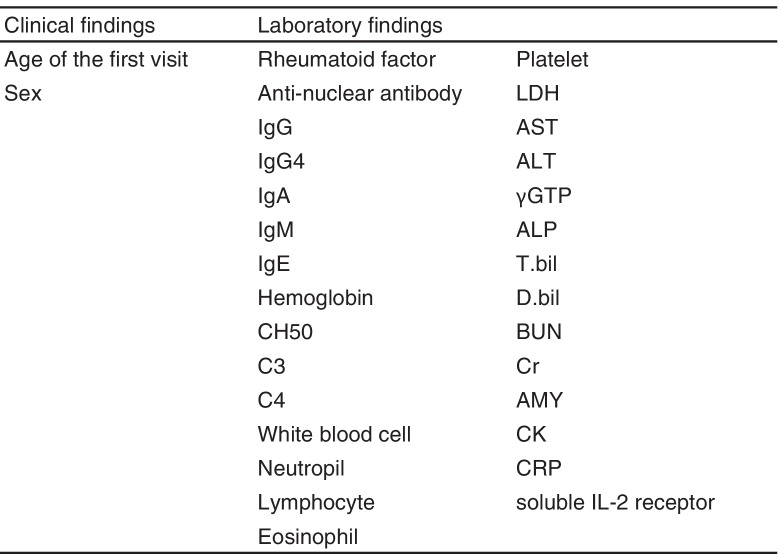
Clinical and laboratory items used for machine learning

CART is a decision tree learner that selects the variable with the highest impurity and performs branching [17]. The random forest method, which is an ensemble learning method, uses the Gini impurity calculated from CART to perform variable selection. The Gini impurity is an indicator of the importance of a variable. The random forest method is used as a practical method when a certain number of samples is available [18]. In addition, both functions visualize the reason for the selection, allowing heuristic knowledge acquisition, which is difficult to imagine from the usual way of handling data.

In terms of model fitting, when the serum IgG4 values were known, the CART method used a cp (a parameter indicating the complexity of the tree model) value of 0.017, and the random forest method used a mtry (the number of variables to be employed in the model) of 5 and a ntree (the number of decision trees to be tried) of 400, and when the serum IgG4 values were unknown, for the CART method used a cp value of 0.015, and random forest method used a mtry of 3 and a ntree of 300 for optimization. The Shapiro-Wilk test was conducted to confirm that the training, test, and validation samples had a normal distribution (*p* = 0.62). Intergroup comparisons were performed using a two-tailed *t* test. The accuracy of the model was retrospectively evaluated by drawing a receiver-operating characteristic (ROC) curve from the sensitivity and specificity of the validation sample, and by calculating the area under the curve (AUC). *P* values less than 0.05 were considered to denote statistical significance. Values are provided as the mean ± standard deviation unless otherwise noted.

## Results

### Patient profiles

Among the 602 patients with IgG4-RD, 350 were male and 252 were female (male:female ratio, 1.4:1). The mean age at the first visit was 64.11 ± 11.46 years. The mean serum IgG concentration was 2204.14 ± 1146.86 mg/dL, and the mean serum IgG4 concentration was 666.00 ± 660.46 mg/dL. Of the 602 patients, 499 had dacryoadenitis and sialadenitis, 192 had autoimmune pancreatitis, 51 had sclerosing cholangitis, 107 had renal involvement, 87 had respiratory involvement, and 182 had retroperitoneal fibrosis; 350 (58.1%) patients had two or more organ lesions (Table 2). In the 204 patients with diseases that needed to be differentiated from IgG4-RD, the male:female ratio was 1:3.9 (42 men and 162 women). The mean age at the first visit was 54.02 ± 16.29 years, which was significantly younger than that in the IgG4-RD group (*p* < 0.001). The details of the diseases are shown in Table 2. Of the patients with non-IgG4-RD, all patients with MCD and EGPA [7, 19] presented with elevated serum IgG4 levels. In the MCD group, the mean serum IgG and IgG4 concentrations were 3828.05 ± 1850.66 and 710.42 ± 1156.82 mg/dL, respectively. No significant difference in the serum IgG4 level was found between the MCD and IgG4-RD groups (*p* = 0.63). e missing data rate was 0.7%.

**Table 2:**
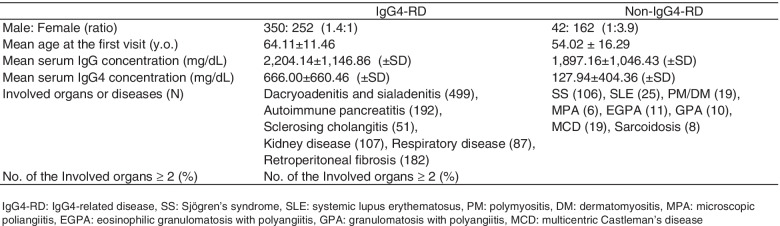
Details of the patients included in the study

### Prediction of the IgG4-RD diagnosis in patients with rheumatic diseases requiring differentiation

**Fig. 1:**
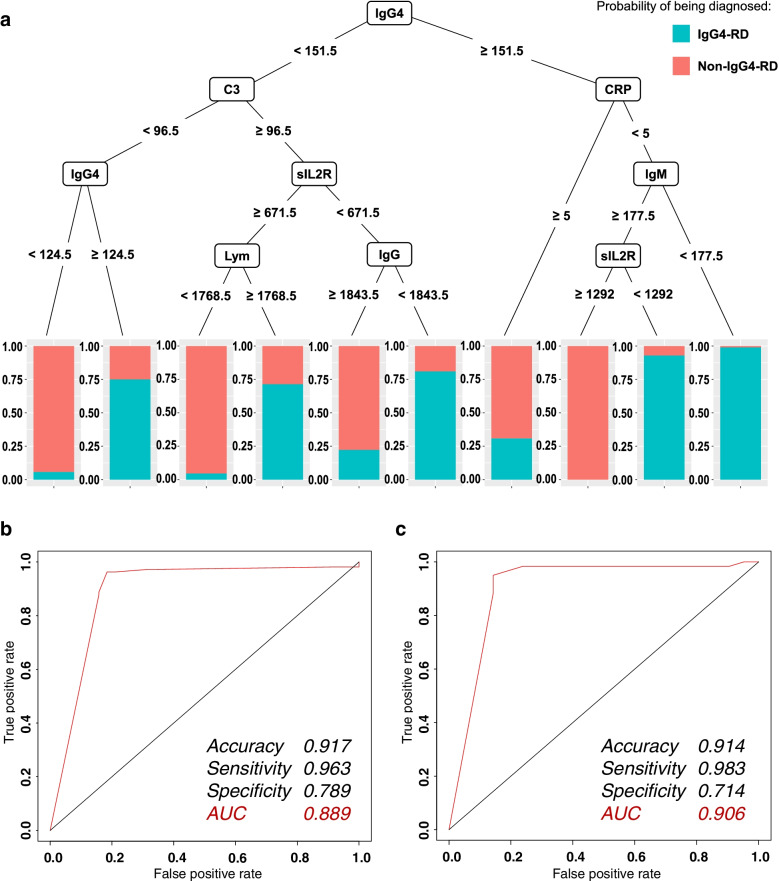
Prediction of IgG4-RD diagnosis in patients with rheumatic diseases requiring differentiation by a CART, when the serum IgG4 level was known. **A** Decision tree algorithm. The blue color in the figure indicates the predicted percentage of IgG4-RD cases, and the red color indicates the percentage of non-IgG4-RD cases. The CART tree model revealed that the key process fluctuations leading to the diagnosis of IgG4-RD in this process were serum levels of IgG4, CRP, IgM, sIL-2R, C3, lymphocyte, and IgG. Furthermore, from top to bottom along the branch to each leaf node of the tree, the “if-then” rules could be generated to predict the diagnosis. For example, the right branch of the CART tree indicated that if serum IgG4 level was ≥151.5 mg/dL, CRP was <5 mg/dL, and IgM was <177.5 mg/dL, it was shown that IgG4-RD is significantly more likely than non-IgG4-RD. **B** ROC curve in the decision tree algorithm (left). The accuracy, sensitivity, and specificity of the algorithm were 0.917, 0.963, and 0.789, respectively, and the AUC was 0.889. **C** ROC curve for the decision tree algorithm (validation) (right). The validation of this algorithm showed that its accuracy, sensitivity, and specificity were 0.906, 0.983, and 0.714, respectively, and the AUC was 0.906

Overall, the data from 806 cases were used in this study; among these cases, 602 were IgG4-RD cases and 204 were non-IgG4-RD cases. The data of 10% of these cases (60 IgG4-RD cases and 21 non-IgG4-RD cases) were retained for validation, and the data of the remaining 725 cases (542 IgG4-RD cases and 183 non-IgG4-RD cases) were used as the training and test samples. When the serum IgG4 level was known, the diagnosis of IgG4-RD was predicted by a decision tree (Fig. 1A). The CART model revealed that the key process fluctuations leading to a diagnosis of IgG4-RD in this process were the serum levels of IgG4, C-reactive protein (CRP), IgM, soluble interleukin-2 receptor (sIL-2R), complement 3, lymphocytes, and IgG. Furthermore, from the top to the bottom along the branch to each leaf node of the tree, “if-then” rules could be generated to predict the diagnosis. For example, the right branch of the CART indicated that if the serum IgG4 level was ≥151.5 mg/dL, CRP was <5 mg/dL, and IgM was <177.5 mg/dL, then IgG4-RD was significantly more likely than non-IgG4-RD. The ROC curve for this algorithm is shown in Fig. 1B. The accuracy, sensitivity, and specificity of the algorithm were 0.917, 0.963, and 0.789, respectively, and the AUC was 0.889. Validation of this algorithm showed that its accuracy, sensitivity, and specificity were 0.914, 0.983, and 0.714, respectively, and the AUC was 0.906 (Fig. 1C).

**Fig. 2:**
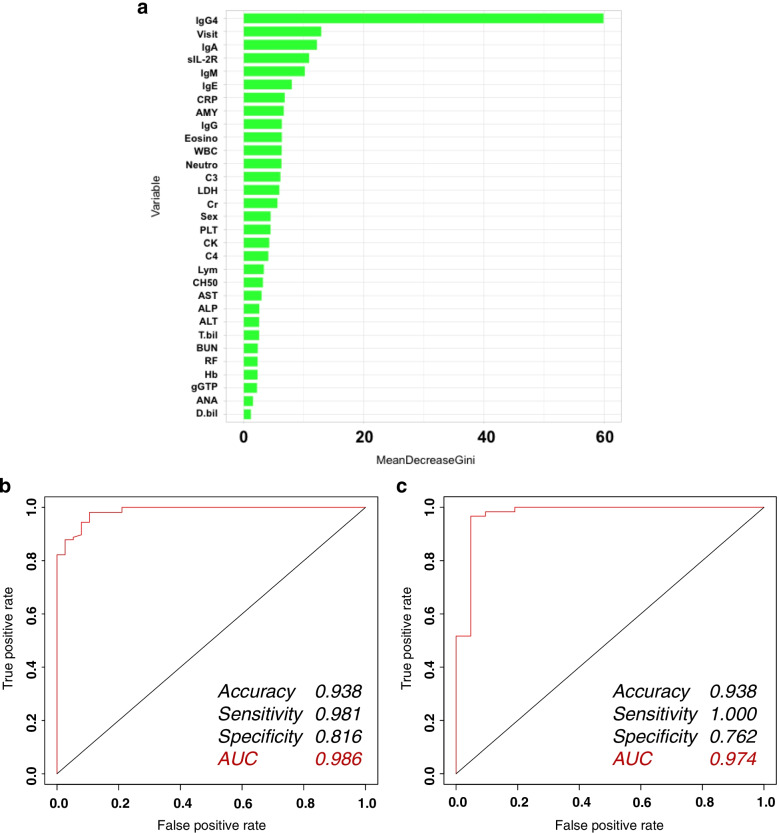
Prediction of IgG4-RD diagnosis in patients with rheumatic diseases requiring differentiation by a random forest, when the serum IgG4 level was known. **A** Decrease in Gini impurity. In this algorithm, the serum IgG4 concentration is the most important variable, followed by the age at the first visit, levels of serum IgA, sIL-2R, and IgM. **B** ROC curve for the random forest algorithm (left). The accuracy, sensitivity, and specificity of the algorithm were 0.938, 0.981, and 0.816, respectively, and the AUC was 0.986. **C** ROC curve for the random forest algorithm (validation) (right). The validation of this algorithm showed that its accuracy, sensitivity, and specificity were 0.938, 1.000, and 0.762, respectively, and the AUC was 0.974

The same data were then analyzed using the random forest method. The Gini impurity is shown in Fig. 2A. In this algorithm, the serum IgG4 concentration was the most important variable, followed by the age at the first visit, and the levels of serum IgA, sIL-2R, and IgM. The ROC curve for this algorithm is shown in Fig. 2B. The accuracy, sensitivity, and specificity of the algorithm were 0.938, 0.981, and 0.816, respectively, and the AUC was 0.986. Validation of this algorithm showed that its accuracy, sensitivity, and specificity were 0.938, 1.000, and 0.762, respectively, and the AUC was 0.974 (Fig. 2C).

**Fig. 3:**
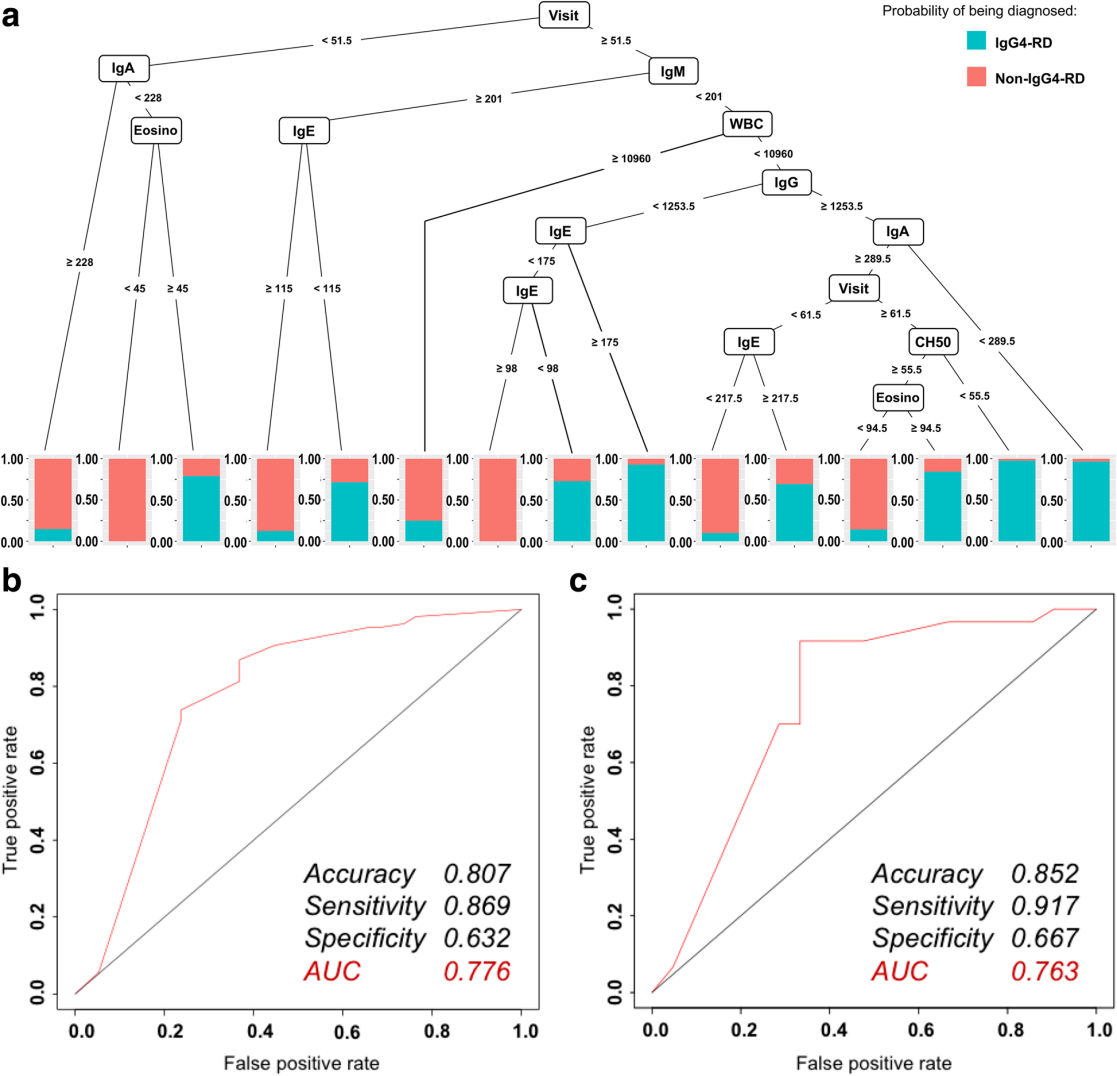
Prediction of IgG4-RD diagnosis in patients with rheumatic diseases requiring differentiation by a CART, when the serum IgG4 level was unknown. **A** Decision tree algorithm. The blue color in the figure indicates the predicted percentage of IgG4-RD cases, and the red color indicates the percentage of non-IgG4-RD cases. The CART tree model revealed that the key process fluctuations leading to the diagnosis of IgG4-RD in this process were the age at the first visit, several serum biomarkers, and the peripheral counts of white blood cells and its fractions. For example, the right branch of the CART tree indicated that if age at the first visit ≥51.5 years, serum IgM level was <201 mg/dL, peripheral counts of leucocytes <10,960/μL, serum IgG level was ≥1,253.5 mg/dL, and serum IgA level was <289.5 mg/dL, it was shown that IgG4-RD is significantly more likely than non-IgG4-RD. **B** ROC curve for the decision tree algorithm (left). The accuracy, sensitivity, and specificity of the algorithm were 0.807, 0.869, and 0.632, respectively, and the AUC was 0.776. **C** ROC curve for the decision tree algorithm (validation) (right). The validation of this algorithm showed that its accuracy, sensitivity, and specificity were 0.852, 0.917, and 0.667, respectively, and the AUC was 0.763

When the serum IgG4 level was unknown, the diagnosis of IgG4-RD was predicted by a decision tree (Fig. 3A). The CART model revealed that the key process fluctuations leading to a diagnosis of IgG4-RD in this process were the age at the first visit, the levels of several serum biomarkers, the peripheral count of white blood cells, and the fractions of the white blood cells. For example, the right branch of the CART indicated that if the age at the first visit was ≥51.5 years, serum IgM level was <201 mg/dL, peripheral count of leukocytes was <10,960/μL, serum IgG level was ≥1253.5 mg/dL, and serum IgA level was <289.5 mg/dL, then IgG4-RD was significantly more likely than non-IgG4-RD. The ROC curve for this algorithm is shown in Fig. 3B. The accuracy, sensitivity, and specificity of the algorithm were 0.807, 0.869, and 0.632, respectively, and the AUC was 0.776. Validation of this algorithm showed that its accuracy, sensitivity, and specificity were 0.852, 0.917, and 0.667, respectively, and the AUC was 0.763 (Fig. 3C).

**Fig. 4:**
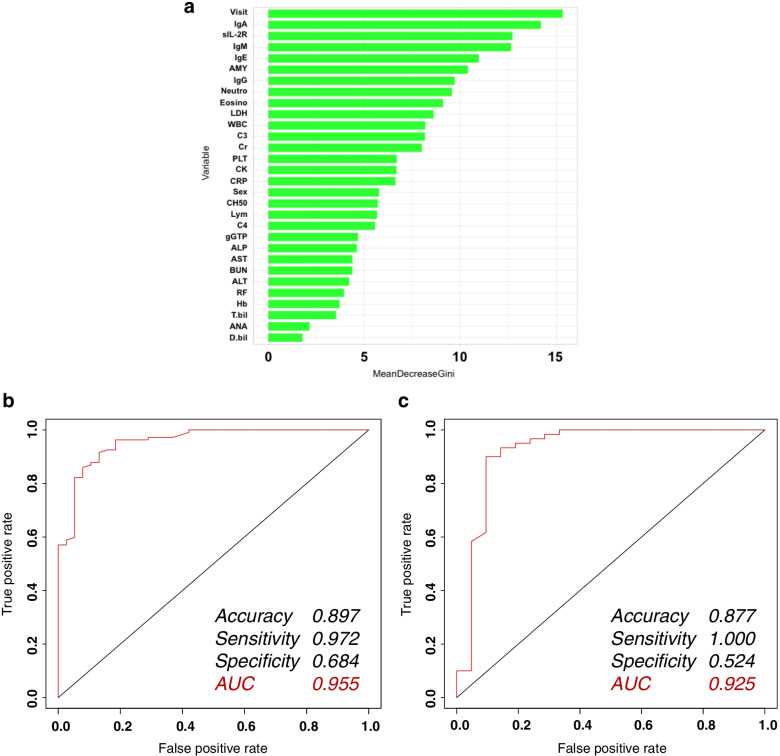
Prediction of IgG4-RD diagnosis in patients with rheumatic diseases requiring differentiation by a random forest, when the serum IgG4 level was unknown. **A** Decrease in Gini impurity. In the Random Forest method, the Gini impurity is an indicator of the importance of a variable. In this algorithm, the age at the first visit is the most important variable, followed by levels of serum IgA, sIL-2R, IgM, and IgE. **B** ROC curve for the random forest algorithm (left). The accuracy, sensitivity, and specificity of the algorithm were 0.897, 0.972, and 0.684, respectively, and the AUC was 0.955. **C** ROC curve for the random forest algorithm (validation) (right). The validation of this algorithm showed that its accuracy, sensitivity, and specificity were 0.877, 1.000, and 0.524, respectively, and the AUC was 0.925

The Gini impurity is shown in Fig. 4A. In this algorithm, the age at the first visit was the most important variable, followed by the levels of serum IgA, sIL-2R, IgM, and IgE. The ROC curve for this algorithm is shown in Fig. 4B. The accuracy, sensitivity, and specificity of the algorithm were 0.897, 0.972, and 0.684, respectively, and the AUC was 0.955. Validation of this algorithm showed that its accuracy, sensitivity, and specificity were 0.877, 1.000, and 0.524, respectively, and the AUC was 0.925 (Fig. 4C).

## Discussion

To our knowledge, this study is the first to construct a machine learning algorithm to differentiate IgG4-RD from non-IgG4-RD based on patient characteristics and laboratory findings.

From the results of the present study, when the serum IgG4 level was included in the analysis, the accuracy was 0.914 for the decision tree method and 0.938 for the random forest method. The AUC of the decision tree method was 0.906, which is above 0.9, and the random forest method had a good accuracy of 0.974. The algorithm of the decision tree depicts the characteristics of this disease very well; in IgG4-RD, more than 90% of the cases have hyper-IgG4emia [7, 20–22], and the CRP level is often low. Yamada et al. reported that less than 10% of cases had a CRP level ≥1.0 mg/dL [23]. Furthermore, the absence of an elevated serum IgM level is considered to be important in differentiating MCD [24]. The most used nodes and branches in this study were diagnosed by CRP, IgM, and hyper-IgG4emia. On the other hand, in the Gini impurity in the random forest method, serum IgG4 was extracted as the most important variable, followed by the levels of serum IgA, IgM, and sIL-2R, and the age at the first visit. The cutoff values for the age at the first visit and serum IgA concentration in the Gini impurity are unknown due to the nature of the random forest method [25], but IgG4-RD often occurs in the elderly [26], and age is presumably used to differentiate IgG4-RD from MCD, as described above [24].

When the serum IgG4 level was not used (as the level was unknown), the accuracy of the decision tree was 0.852, and the AUC was 0.763, which is much lower than when the IgG4 level was known, but the random forest method was able to obtain a good diagnostic prediction of 0.925. The algorithm of the decision tree showed that it is more complicated when the IgG4 value is unknown than when it is known. Even the most frequently used branch of the tree had five nodes: they were the age at the first visit, the concentrations of serum IgG, IgA, and IgM, and the peripheral leukocyte counts. In this tree, a new node was added: a peripheral white blood cell count <10,960 /μL. The peripheral white blood cell count has not been given much attention in the diagnosis of IgG4-RD until now. In the future, as a new perspective, it may be necessary to keep in mind that leukocytosis is not very common in IgG4-RD. In contrast, in the Gini impurity in the random forest method, in which the accuracy was restored, the age at the first visit was extracted as the most important variable. Regardless of whether the serum IgG4 level was known, the most important factors that were extracted were generally the same. In other words, in the random forest method, when the serum IgG4 level is known, it is the most important factor for diagnosis, and the age at the first visit and the levels of serum Ig and sIL-2R are also important variables, and when the serum IgG4 level is unknown, the age at the first visit and serological markers other than the serum IgG4 concentration are important for the diagnosis.

There are several limitations in this study. Even though this study is a multicenter study, the amount of data analyzed is still considered small. When building a machine learning model, a large representative and diverse dataset should be collected. Since this study focused on the differential diagnosis of IgG4-RD, it was not adjusted to the frequency of the disease in actual clinical practice. In addition, all subjects were Japanese, and it is unclear whether our results can be extrapolated to other populations. Also, the test items used in this study included items that are not used in daily practice in Europe and the USA. In Japan, registries for intractable diseases are currently being constructed, and we hope to overcome these problems in the future using the databases from these registries and through collaborations with other researchers throughout the world. Machine learning is expected to be widely used in lifelong health management [27], and in the near future, we will build a large database that will be constantly updated and close to daily clinical practice. In addition, we would like to integrate the clinical and multi-omics data to establish an algorithm that can be applied for the diagnosis of IgG4-RD and other diseases, and for the prediction of prognoses, such as for drug selection, and responses to treatment. Based on the results of this study, we believe that AI will facilitate the further understanding of pathological conditions and enable drug discovery.

## Conclusions

Based on our investigation of machine learning in a multicenter collaboration, we found that with or without serum IgG4 data, basic patient characteristics, and blood test findings alone were sufficient to differentiate IgG4-RD from non-IgG4-RD. When the serum IgG4 level was known, it was the most important factor for the diagnosis, and the age at the first visit and the concentrations of Ig and sIL-2R were also important variables. Even in cases in which the serum IgG4 concentration was unknown, the age at the first visit and the concentrations of Ig and sIL2R were important in the diagnosis.

## Data Availability

All data generated or analyzed during this study are included in this published article.
